# Receptor-Drug Interaction: Europium Employment for Studying the Biochemical Pathway of G-Protein-Coupled Receptor Activation

**DOI:** 10.1155/2007/12635

**Published:** 2008-02-25

**Authors:** Colabufo Nicola Antonio, Perrone Maria Grazia, Contino Marialessandra, Berardi Francesco, Perrone Roberto

**Affiliations:** Dipartimento Farmacochimico, Facoltá di Farmacia, Universitá degli Studi di Bari, via Orabona 4, 70125 Bari, Italy

## Abstract

In medicinal chemistry field, the biochemical pathways, involved in 7-transmembrane domains G-protein coupled receptors (GPCRs) activation, are commonly studied to establish the activity of ligands towards GPCRs. The most studied steps are the measurement of activated GTP-α subunit and stimulated intracellular cAMP. At the present, many researchers defined agonist or antagonist activity of potential GPCRs drugs employing [^35^S]GTP*γ*S or [^3^H]cAMP as probes. Recently, the corresponding lanthanide labels Eu-GTP and Eu-cAMP as alternative to radiochemicals have been developed because they are highly sensitive, easy to automate, easily synthesized, they display a much longer shelf-life and they can be used in multilabel experiments. In the present review, the receptor-drug interaction by europium employment for studying the biochemical pathway of GPCR activation has been focused. Moreover, comparative studies between lanthanide label probes and the corresponding radiolabeled compounds have been carried out.

## 1. INTRODUCTION

**Figure 1:**
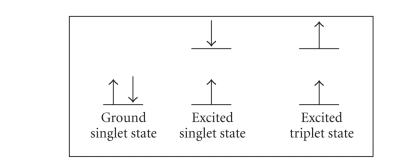
*Singlet* states (all electrons in the molecule are spin-paired) and *triplet* states (one set of electron spins is unpaired) in excited molecules.

Luminescence is defined as emitted radiation by a chemical species, molecule, or atom. It occurs when an electron returns to the electronic ground state from an excited state, loosing its excess energy as a photon. Electronic states of organic molecules can be grouped into *singlet* and *triplet* state. The *singlet* state is characterized by the presence of all the electrons with their spins paired, while the *triplet* state displays one set of the electron spin unpaired (see Figure 1).

**Figure 2:**
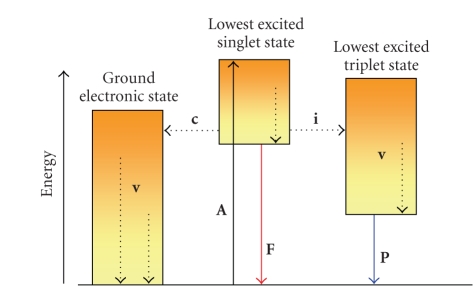
Possible physical process following absorption of a photon by a molecule; A = absorption; F = fluorescence; P = phosphorescence; *processes involving photons * = continue arrows; * Radiationless transitions* (v = vibrational relaxation, i = intersystem crossing, c = internal conversion) = dotted arrows.

The phosphorescence and fluorescence light emissions are the main phenomena belonging to luminescence (see Figure 2).

Phosphorescence occurs when the excited electron in the *singlet* state undergoes intersystem crossing to a metastable *triplet* state and then to the electronic ground state with the emission of a photon. Since this phenomenon originates from the lowest *triplet* state, it shows a decay time approximately equal to the lifetime of the *triplet* state (from 10^−4^ to 10 seconds). Therefore, phosphorescence is often characterized by an afterglow which is not observed for fluorescence.

Fluorescence occurs when the molecule returns to the electronic ground state from the excited *singlet* state by emission of a photon and the lifetime of an excited *singlet* state is approximately from 10^−9^ to 10^-7^ seconds. To date, many biological and medical applications are based on fluorescence properties of pharmacological tools. These compounds permit to visualize single step activated in cell biochemical pathways, to estimate changes in functional and structural property cells, and to appreciate the modification of their molecular complexes involved in biological systems.

Indeed, the fluorescence spectroscopy is one of the most important applications for monitoring the molecular interactions at the single molecule level and now widely used in biological research.

In particular, the receptor-ligand interaction study, which represents the starting point of drug discovery in medicinal chemistry field, could be better performed by fluorescence resonance energy transfer (FRET) and bioluminescence resonance energy transfer (BRET) techniques.

At the present, this study is carried out employing radioligands that display some limitations due to the safety, the storage, the handling, and the disposal of radioactive materials; other limitations are due to the difficulties to perform analysis in living cells. The development of lanthanide-based assays to assess receptor-ligand interactions is improving the characterization and the evaluation of potential new drugs and pharmacological tools discovery [1–3].

Lanthanide labels are an alternative to radiochemicals because they are highly sensitive, easy to automate, easily synthesized, they display a much longer shelf life, and can be used in multilabel experiments.

In the present review, the receptor-drug interaction by europium employment for studying the biochemical pathway of GPCR activation will be focused.

**Figure 3:**
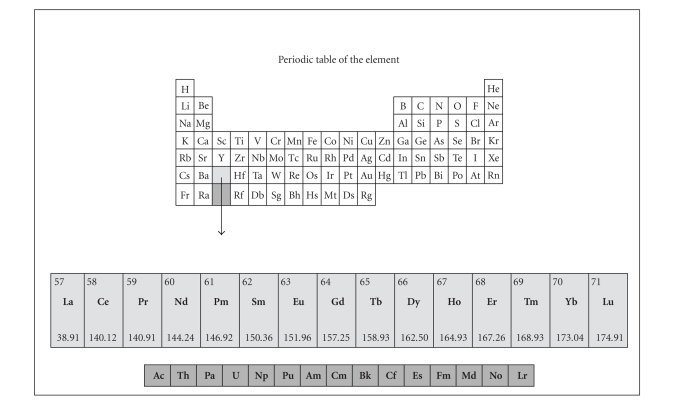
Periodic table of the elements visualising lanthanides.

The lanthanide series comprises the 15 elements with atomic number 57 through 71 as depicted in Figure 3. The complexes of some lanthanides such as Samarium (Sm^3+^), Europium (Eu^3+^), Terbium (Tb^3+^), and Dysprosium (Dy^3+^) are known to be luminescent; their ions emit fluorescent light of specific wavelengths when coordinated to specific ligands. The lanthanide ions in solutions or in complex possess luminescence properties because of the transitions within 4f-shell. The excitation of lanthanide ions occurs at the expense of the intramolecular energy transfer from excited organic molecule to lanthanide ion. The sensitization of luminescence of lanthanide ions in complexes with organic ligand allows their application as luminescence probes to establish the structure and the properties of biological objects.

The major advantages of lanthanide labels are (i) ultrasensitive and specific signal, (ii) low background, (iii) amenability to automation, as well as (iv) stability and safety.

The specific signal from the lanthanide is due to the long lifetime of the excited state that can be temporally separated from the nonspecific signal [1–5]. Typical lifetimes of background fluorescence from plates, reagents, or cells are ranging from picoseconds to microseconds [6, 7] while lanthanide lifetimes are from 0.2 to 1.5 milliseconds. Thus, the excited state of lanthanides is long lived. This gives the advantage of being able to excite the label, to delay measurement of the emission signal until the background fluorescence has completely decayed, and then to collect the specific emission signal from the lanthanide. This delay period leads to an ultrasensitive and specific signal which can be time averaged.

Lanthanides excitation occurs in the ultraviolet region while the emission is in the visible spectrum as depicted in Table 1.

**Figure 4:**
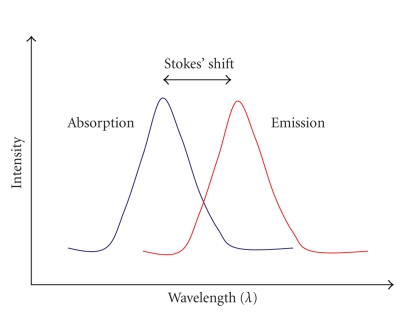
Stokes’ shift.

The emitted light is at a longer wavelength (lower energy) than the absorbed light since some of the energy is lost because of the vibrations; the difference in wavelenght between positions of the band of the excitation and emission is termed *Stokes’ shift* as depicted in Figure 4. The large Stokes’ shift (greater than 200 nm) of the lanthanide ions contributes to the low background signal since there is minimal crosstalk between excitation and emission signals. In addition, the emission peak is very sharp allowing tight limits for the excitation filter sets. These features make this method amenable to use with multiple labels, since Eu, Sm, Dy, and Tb have different excitation and emission profiles and different decay times. Hence, multiple assays can be performed in a single well, thus greatly reducing the number of time and reagents needed. Finally, not less important is the safety of fluorescent probes instead of radiolabeled probes. Indeed, fluorescent probes do not have the drawbacks of radioactivity such as the production, delivery, and disposal of the radioactive materials; the relatively short shelf life of some radionuclides; and the long signal acquisition times required to reach the desired sensitivity.

To detect lanthanides by time-resolved fluorescence (TR- F) in biological assays, a sensitization is necessary. For this purpose, organic chromophores are covalently attached to the lanthanide chelate. The chromophore acts as an antenna which absorbs light. This energy is transferred to the lanthanide excited state and emitted as a fluorescent signal. Since water molecules will deactivate the lanthanide ion, an ideal chelator will saturate the coordination shell of the lanthanide to prevent water from binding.

The lanthanide chelators are divided into two groups: photoactive and nonphotoactive subclasses. A photoactive chelator is useful because firstly, it provides the attachment of the chromophore to the lanthanide facilitating energy transfer; secondly, it protects the lanthanide from coordination with water, and it permits the attachment of other reactive groups [8]. An alternative approach is to use nonphotoactive chelators which should be stable, hydrophilic, and capable of releasing the lanthanide after the addition of an enhancement solution because in this case the fluorescent chelator is contained within this solution.

**Figure 5:**
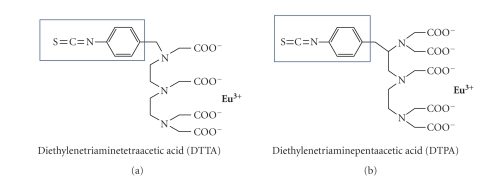
Eu^3+^ stable complexes with DTTA and DTPA.

The most common chelators used in monitoring ligand-receptor interactions are Eu-chelates of diethylenetriaminetetraacetic acid (DTTA) and diethylenetriaminepentaacetic acid (DTPA). These chelates, used for protein labeling, bear an isothiocyanate group which reacts with the *ε*-amino lysine residues. As depicted in Figure 5, DTTA and DTPA form a stable complex with Eu^3+^ by their four and five carboxylic acid groups, respectively [9].

In most assays, to obtain a measurable TRF signal, the lanthanide must be released from the nonphotoactive chelator and must be transferred to a fluorescent chelator contained within the enhancement solution.

## 2. LANTHANIDES APPLICATIONS

The lanthanide chelates have found applications in biomedical assays starting from radiotherapy based on some samarium isotopes, nuclear magnetic imaging, specific cleavage of DNA or RNA, and for sensing of various analytes and conditions. Europium chelates have been used for sensing several determinations, such as, pH [10], temperature [11], light doses, phosphate with unsaturated europium chelate [12], glucose by time-resolving imaging [13], catalase by using tetracycline as enhancing ligand [14], neurotoxic agents for bioterror monitoring [15], and anesthetic agents using a dried strip reagent [16].

The photoluminescence of lanthanides is largely applied in bioanalytical assays especially in the field of clinical immunodiagnostic and for evaluating receptor-ligand interactions.

Two of the most frequently applied technologies for these purposes are the dissociation-enhanced Lanthanide Fluorescent ImmunoAssay (DELFIA), a heterogeneous assay technology based on fluorescence enhancement from PerkinElmer and homogeneous Time Resolved-Fluorescence Resonance Energy Transfer (TR-FRET) (TRACE) from Brahms.

DELFIA has been used for the analysis of different biochemical pathways by monitoring second messengers (calcium, cAMP) for the study of G-protein and kinases activation and for cell viability determination.

## 3. EVALUATION OF BIOLOGICAL PATHWAYS INDUCED BY RECEPTOR-DRUG INTERACTION

**Figure 6:**
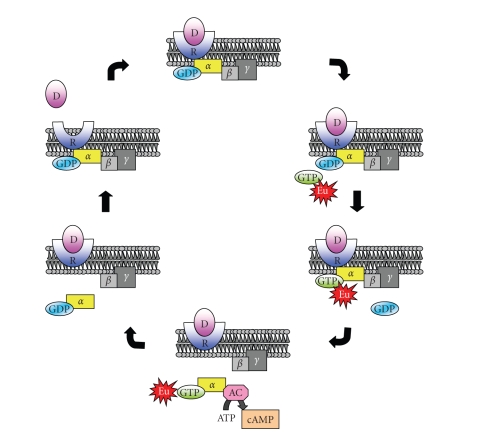
GPCRs activation cycle.

The interaction between G-protein-coupled receptors (GPCRs) with drugs activates specific cell pathways. GPCRs, also known as seven transmembrane receptors (7-TM re ceptors), are a large family of eukaryotic transmembrane receptors that activate several pathways and are involved in many diseases. GPCRs are characterized by three subunits *α*, *β*, and *γ*; and the *α* subunit, in the nonactivated state, binds GDP. GPCR stimulation by agonists leads to the dissociation from the *α* subunit of the GDP and to its replacement with GTP. This GTP-G_*α*_ complex detaches from the *β* *γ* subunit and both of these complexes can lead to downstream signaling. These activities are modulated in cells by GTPase activity that allows the G-protein subunit to return to its inactivated GDP form, hydrolyzing the GTP as depicted in Figure 6.

The first biochemical step is monitored by quantifying the amount of GTP associated with the membrane. To date, the amount of GTP produced by GPCR agonist activation has been evaluated by binding experiment using [^35^S]GTP*γ*S. Nowadays, several research groups start to use fluorescent probes to phase out radioactivity-based methods.

DELFIA GTP-binding assay is directed to the measurement of GPCRs activation in membrane preparations employing a nonhydrolyzable GTP-Eu-label. This method is a time-resolved fluorometric assay based on GDP-GTP exchange on G*α* subunit followed by agonists GPCR activation.

As shown in Table 2, (-)-epinephrine activates *α*_2A_-adrenergic receptor (AR), displaying EC_50_ = 4.7 nM and EC_50_ = 25 nM in the Eu-GTP and [^35^S]GTP*γ*S assays, respectively [17]. Superimposed results were obtained by (-)-epinephrine in *β*_2_-AR binding experiments (EC_50_ = 65 nM and EC_50_ = 67 nM, in the Eu-GTP and [^35^S]GTP*γ*S assays, resp.).

Engström et al. reported that the stimulation of neuropeptide FF receptor (NPFF_2_) by (1DMe)Y8Fa resulted in binding of GTP to the receptor with EC_50_ = 6 nM for Eu-GTP and EC_50_ = 17 nM for [^35^S]GTP*γ*S [18]. Quinpirole, full agonist at dopamine D_3_ receptors, displayed the same potency (EC_50_ = 25 nM) in both the assays [19, 20].

These results suggest that the Eu-GTP binding assay is a reasonable alternative to the traditional [^35^S]GTP*γ*S binding assay, and that lanthanides are replacing radiolabeled traditional methods to characterize the biological pathways linked to receptor-drug interactions.

For a more wide application of Eu-GTP assay, this method should be studied on several GPCRs subtypes.

**Figure 7:**
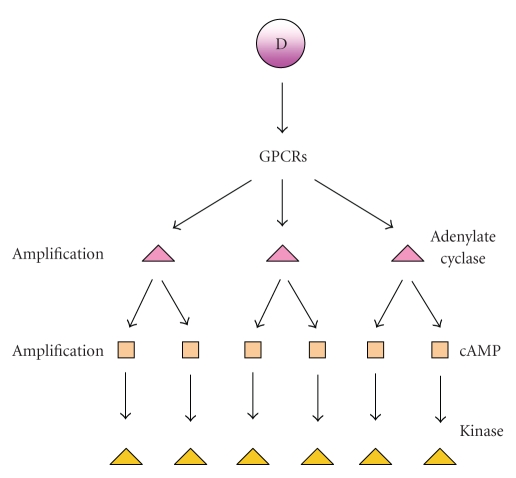
Signal amplification in GPCRs activation.

Eu-GTP and [^35^S]GTP*γ*S assays are usually used on cloned cell lines and this is an advantage with respect to the use of animal tissues. The employment of cell lines in some cases constitutes a limit because of the low receptor expression and/or the presence of other GPCR system quenching the signal. More specific and amplified signals (see Figure 7) can be obtained detecting cyclic AMP (cAMP) or kinases on living cells.

**Figure 8:**
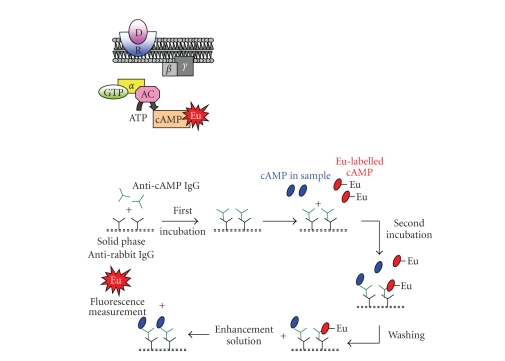
cAMP assay by europium measurement.

cAMP is an important second messenger mediating several physiological responses of neurotransmitters, hormones, and drugs. cAMP is formed by ATP, and its intracellular concentration is regulated by two membrane-bound enzymes: adenylate cyclase (AC) and phosphodiesterase. The cAMP concentration in cells is stimulated by the activation of adenilate cyclase, responsible for the ATP conversion into cAMP upon ligand binding to GPCR (see Figure 8). The first method directly measuring the cAMP levels is based on radioisotopes using scintillation proximity assay (SPA) tecnology [21–24]. An alternative method is DELFIA. This assay is intended for the quantitative determination of cAMP in cell-culture samples. This method is a solid-phase time-resolved fluoroimmunoassay based on the competition between europium-labeled cAMP and cAMP of the samples for the binding sites of cAMP-specific polyclonal antibodies from rabbit. A second antibody, directed against rabbit IgG, is coated to the solid phase, allowing the separation of the antibody-bound and the free antigen. The addition of an enhancement solution to each sample permit the dissiociation of the europium ions from the labeled antigen into solution, where they form highly fluorescent chelates with the components of enhancement solution (see Figure 8). The fluorescence detected is inversely proportional to the amount of cAMP in the sample.

Eu-cAMP constitutes a suitable alternative to the common radiolabeled method, [^3^H] cAMP, commonly used to evaluate the accumulation of cAMP in cell line overexpressing *β*_3_-AR.

As listed in Table 3, EC_50_ values for three reference compounds, isoproterenol, epinephrine, and norepinephrine on *β*_3_-AR, by using [^3^H]cAMP [25] are superimposed (EC_50_ = 3.9, 49, and 6.3 nM, resp.) with those obtained with DELFIA (EC_50_ = 5.8, 31, and 5.5 nM, resp.) [26].

These results suggest that also in this case, the Eu-cAMP binding assay is a reasonable alternative to the traditional radiolabeled binding assay.

Moreover, fluorescent probes have also been developed to study the activity of some protein kinases. The protein kinases are a class of enzymes classified as PKC*α*, PKC*β*, and PKC*γ*, each having a specific function. These enzymes remove a phosphate group from ATP and covalently attach it to one of the three aminoacids having a free hydroxyl group (serine, threonine, and tyrosine) chemically modifying other proteins. This phosphorylation usually results in a functional change of the target protein. In this way, kinases regulate the majority of cell pathways involved in signal transduction. Protein kinases are turned on or off by phosphorylation (sometimes by autophosphorylation) by binding of activator or inhibitor proteins. Disregulation of kinases activity is a frequent cause of several diseases such as cancer because kinases regulate many aspects that control the cell growth, movement, and death. Currently, several quantitative and sensitive nonradioactive in vitro assays such as DELFIA for monitoring kinases activity and kinases phosphorylation are reported in literature. Herein, the detection of Nek2, Insulin receptor, and IKK complex is elicited.

Never In Mitosis Arrest- (NIMA-) related kinase 2, also known as Nek2, is a serine/threonine kinase required for centrosome splitting and bipolar spindle formation during mitosis. Nek2 phosphorylates a large centrosomal linker protein called c-NAP1 (centrosomal Nek2-associated protein 1), which subsequently triggers the release and separation of duplicated centrosomes [27]. Nek2 has been demonstrated to be concentrated primarily in the centrosomes of rapidly proliferating cells. High expression of Nek2 compared to normal tissue has been observed in lung, colon, and breast carcinomas as well as B-cell lymphomas. Downregulation of Nek2 and overexpression of a kinase-defective dominant negative enzyme result in (i) lack of centrosome separation, (ii) defective spindle formation, (iii) increased apoptosis, and (iv) decreased cell proliferation. Thus, targeting Nek2 in tumour cells by a small molecule inhibitor may have the same phenotypic consequences. For this kinase, two different DELFIA assays have been developed. One method uses a peptide identified within c-NAP1, the other one employs Nek2 enzyme as a substrate to monitor autophosphorylation.

DELFIA resulted in a useful assay to monitor changes in kinases phosphorylation instead of the traditional measure of radioactive phosphate incorporation or the use of phosphokinase antibodies (ELISA and Western blot). For example, the insulin receptor (IR) is a tyrosine kinase composed of two extracellular *α* subunits and two transmembrane *β* subunits. Insulin action starts by its binding to the *α* subunits of the IR, and causes conformational changes that lead to autophosphorylation of the *β* subunits and activation of the receptor tyrosine kinase [28]. Moreover, DELFIA assay can be easily adapted to monitor changes in the phosphorylation status of other cellular proteins.

IKK complex consists of two kinases, IKK*α* and IKK*β*, and of the regulatory nonenzymatic scaffold protein IKK*γ* also known as NEMO (NF-*κ*B essential modifier) [29–33]. I*κ*B phosphorylation by IKK results in the degradation of I*κ*B, allowing NF-*κ*B to translocate to the nucleus and activate transcription of a variety of genes. Many studies have indicated that activity of IKK*β* is directly related to TNF*α* activation, whereas IKK*α* is critical for the development of the skin and skeleton during embryogenesis. To elucidate the mechanisms by which NF-*κ*B is activated, it is important to have an effective assay to examine different pathways. Traditionally, IKK activity has been measured by a radioactive kinase assay utilizing [^33^P]ATP or [^32^P]ATP as a donor. For monitoring substrates phosphorylation, DELFIA resulted in sensitive and efficient nonradioactive assays to detect multiple kinase activities simultaneously, including IKK [34].

## 4. CONCLUSIONS

Lanthanide labels constitute a perspective in medicinal chemistry field for studying the activity of potential new drugs towards GPCRs. To date, few results relating to Eu-GTP and Eu-cAMP and comparable to the corresponding radiolabeled probes are available. With respect to radiolabeled method, these new tools display several advantages such as the endocellular pathways investigation in living cells, the high sensitivity, the long shelf life, the amenability to automation, the safety, and last but not least, they represent the novel green biochemistry. At the present, it is important to investigate Eu-GTP and Eu-cAMP impact in several GPCRs in order to better define the potentials of lanthanide labels in medicinal chemistry field.

## Figures and Tables

**Table 1:** Lanthanides spectroscopic data.

Lanthanide	*^λ^* *excitation* (nm)	*^λ^* *emission* (nm)	Visible spectrum region
Tb	320	545	Green
Dy	320	572	Yellow
Eu	340	615	Red
Sm	340	642	Red

**Table 2:** Eu-GTP and [^35^S] GTP*γ*S assays comparison.

Receptor subtypes	Reference compounds	Eu-GTP	[^35^S]GTP*γ*S
		EC_50_, *n*M	
*α*_2__A_	Epinephrine	4.7	25
*β*_2_	Epinephrine	65	67
NPFF_2_	(1DMe)Y8Fa	6.0	17
D_3_	Quinpirole	25	25

**Table 3:** Eu-cAMP and [^3^]cAMP assays comparison.

	[^3^H]cAMP	Eu-cAMP
	EC_50_, nM	
Isoproterenol	3.9	5.8
Epinephrine	49	31
Norepinephrine	6.3	5.5
